# The Effect of Different Final Irrigation Regimens on the Dentinal Tubule Penetration of Three Different Root Canal Sealers: A Confocal Laser Scanning Microscopy Study *In Vitro*

**DOI:** 10.1155/2021/8726388

**Published:** 2021-10-14

**Authors:** Tufan Ozasir, Birgul Eren, Kamran Gulsahi, Mete Ungor

**Affiliations:** ^1^Department of Endodontics, Faculty of Dentistry, Baskent University, Ankara, Turkey; ^2^Department of Endodontics, Faculty of Dentistry, Medipol University, Istanbul, Turkey

## Abstract

This study evaluated the effects of different final irrigation regimens on the dentin tubule penetration of three different root canal sealers using confocal laser scanning microscopy (CLSM). A total of 160 single-rooted extracted mandibular premolar teeth were divided into five groups (*n* = 32 each) according to the solution used in the final rinse protocol, as follows: 17% ethylenediaminetetraacetic acid (EDTA) (group 1), 17% EDTA and 2% chlorhexidine gluconate (CHX) (group 2), 7% maleic acid (MA) (group 3), 7% MA and 2% CHX (group 4), and 5.25% NaOCl (group 5). Two roots from each group were examined under scanning electron microscopy (SEM) to visualize smear layer removal. Experimental groups were then split randomly into three subgroups (*n* = 10) and obturated using a cold lateral condensation technique with 0.1% rhodamine B-labelled sealers [either AH Plus (group A), EndoREZ (group E), or Tech BioSealer Endo (group T)] and gutta-percha. Specimens were sectioned and observed by CLSM to evaluate the percentage and maximum depth of sealer penetration at the apical, middle, and coronal levels. Statistical comparison was performed on grouped (apical, middle, and coronal segments) and ungrouped data using two-way ANOVA with Bonferroni post hoc test (*p* < 0.01). AH Plus showed the greatest tubule penetration while Tech BioSealer Endo showed the least. Resin-based sealers displayed deeper and more consistent penetration. CHX irrigation positively influenced sealer tubule penetration.

## 1. Introduction

The key objective of root canal therapy is to remove microorganisms out of the root canal system to prevent recontamination. However, due to the root canal system's complex anatomical structure, it is impossible to ensure complete cleaning of the root canal using only instrumentation methods [1]. Microorganisms may be present in the root canal even after biomechanical procedures [2, 3]. The purpose of filling operations is to ensure proper filling of the root canal system, along with accessory canals, apical delta, isthmus, and dentinal tubules. Sealer cement within dentinal tubules entombs any residual microorganisms keeping them distant from nutritional sources. Deep penetration of endodontic sealer also diminishes the interface area between root dentin and the filling material and might improve retaining of the filling mass due to locking in a mechanical fashion [4, 5]. Root canal instrumentation results in a smear layer containing bacteria and debris, including necrotic tissue debris [6]. This smear layer adheres to the surface of dentin and occludes the dentinal tubules. It therefore prevents irrigant solutions, medication, and root canal sealers from penetrating these dentinal tubules and must be removed [7]. Solutions used for this purpose include sodium hypochlorite (NaOCl), maleic acid (MA), ethylenediaminetetraacetic acid (EDTA), citric acid, and phosphoric acid. Ideally, an endodontic irrigant is required to dissolve both the organic matter and the inorganic matter of the smear layer within the root canal [8, 9]. NaOCl is commonly used during endodontic treatment. However, despite its strong activity in antibacterial terms, NaOCl is ineffective on the smear layer's inorganic component. Therefore, it is most common to use NaOCl in conjunction with EDTA, which dissolve the smear layer's organic and inorganic segments, respectively [4, 10]. As a mild organic acid, MA has lately been suggested as an alternative solution for irrigation owing to its capability of removing the smear layer and its reduced toxicity, where maleic acid further has antibacterial properties which can eradicate *Enterococcus faecalis* biofilms in comparison to EDTA or citric acid [11].

Chlorhexidine gluconate (CHX) is also employed as a root canal irrigant because of its broad-spectrum antibacterial activity against both the Gram-positive and the Gram-negative bacteria and facultative anaerobic and aerobic bacteria, as well as viruses, yeast, and spores. However, its inability to dissolve organic tissues is a major shortcoming. The activity of CHX is pH-dependent and is dramatically lowered in the existence of organic matter [12, 13].

Dentinal tubule penetration of endodontic sealants is dependent on their physical and chemical qualities, including surface tension, viscosity, solubility, and particle size [14]. EndoREZ (Ultradent Products, Inc., South Jordan, UT) is a first-generation hydrophilic urethane dimethacrylate dually cured bondable endodontic root canal sealant with resin base which requires no extra dentin adhesive [15].

Tech Biosealer Endo (Isasan, Rovello Porro, Italy) is a calcium silicate-based endodontic sealer with improved antibacterial effect, impeccable biocompatibility, and outstanding apical sealing as well as radiopacity [16].

An epoxy root canal filling sealant with resin base, AH Plus (Dentsply Maillefer, Ballaigus, Switzerland), is qualified as an excellent example among other sealants since it can be well adapted to root canal walls and has outstanding physical qualities [17].

Confocal laser scanning microscopy (CLSM) is an excellent tool for evaluation of dentinal tubule penetration on account of its ability to produce standardised, reproducible 3-D imaging, and the detailed information it provides at lower magnifications (10x) by means of fluorescent rhodamine-marked sealants [10, 15, 18].

This ex vivo study is aimed at evaluating the effects of different regimens of final irrigation on the dentinal tubule penetration of three different root canal sealers using CLSM.

## 2. Materials and Methods

For this study which was approved by the Institutional Review Board of Baskent University (project no: D-DA 14/03), 160 human mandibular premolar teeth having a single, straight root and canal but no apical resorption, completely formed root apexes and having a distance of 14 mm from cervical margin to root apex, were selected. The root surface was cleared of the soft tissue remnants, and teeth were kept with distilled water till their use. The teeth were all decoronated below the cementoenamel junction using a diamond bur with water cooling at low speed (Diatech Swiss Dental Instruments, Altstätten, Switzerland). To define the working length, a size 10 K file was inserted until it became apparent at the apical foramen, followed by subtracting 1 mm from the total length of the root canal.

Biomechanical preparation was performed as follows: the coronal aspect of the root canal was flared using Gates Glidden drills of size 2 and size 3, and then, the apical portion was enlarged up to a NiTi K-file of size 40 (Dentsply Maillefer). Subsequently, step-back preparation was done up to a NiTi K-file of size 55. During cleaning and shaping, 1 mL of 5.25% NaOCl and a 27-gauge irrigation needle were used to irrigate the canals at every instrument change. Based on the solution employed in the latest rinse process, the roots were all randomly segregated into five groups (*n* = 32) which include the following:

- Group 1: 5 mL 17% EDTA (Sigma-Aldrich, Taufkirchen, Germany) for 60 sec and 2.5 mL distilled water
- Group 2: 5 mL 17% EDTA for 60 sec, 2.5 mL distilled water, and 2.5 mL 2% CHX (Drogsan, Ankara, Turkey) for 60 sec
- Group 3: 5 mL 7% MA (Merck Chemistry, Darmstadt, Germany) for 60 sec and 2.5 mL distilled water
- Group 4: 5 mL 7% MA for 60 sec, 2.5 mL distilled water, and 2.5 mL 2% CHX for 60 sec
- Group 5: 5 mL 5.25% NaOCl for 60 sec and 2.5 mL distilled water

For the analysis of smear layer removal, two roots from each group were made ready to get scanned using an electron microscopy (SEM) (Quanta 200 FEG). The teeth that remained in all groups were randomly assigned into three subgroups (*n* = 10) based on the sealant to be employed: Group A—AH Plus (A), Group E—EndoREZ, and Group T—Tech BioSealer Endo.

For observation by a confocal laser scanning microscope (CLSM), all sealants were blended together with 0.1% fluorescent rhodamine B isothiocyanate dye (Merck Chemistry). Finally, paper points were used to dry all canals (Sure-endo, SureDent), and then, the sealant was inserted into the root canals until 1 mm remains to the working length by using lentulo spirals (TGdent) of size 25 at a low speed. A standardised master gutta-percha cone (size 40) with tug-back was placed inside the canal up to the working length, followed by performing obturation by means of a lateral condensation technique. The level of quality of the root canal fillings performed was verified through radiography. The access cavity was made tight using a transient filling material (Cavit G, 3M ESPE, USA), and then, the specimens were kept at 37°C under 100% humidity for seven days for the sealer to set. All specimens were cut at a right angle to the long axis by means of a diamond disc under constant water refrigeration. Horizontal sections (1 mm thickness) were cut 1 mm, 5 mm, and 9 mm from the apical tip, and the surfaces of the sections were polished with a sandpaper. Samples were mounted on glass slides and examined under a CLSM (Zeiss LSM 510, Carl Zeiss, Göttingen, Germany) to evaluate the depth of sealer penetration. At 512 × 512 pixels and 10x magnification, images were treated from one focal plane. Laser argon excitation was attained at 514 nm. Image analysis was performed using Zeiss LSM Image Browser (Carl Zeiss Micro Imaging, GmbH, Germany).

## 3. Statistical Analysis

Data were analyzed using SPSS 18 software (SPSS Inc., Chicago, IL, USA). Continuous variables were subjected to the Shapiro-Wilk test for normality, and the Levene test was used for homogeneity of variances. The percentage of tubule penetration and maximum penetration depth scores between groups was examined by two-way variance analysis. To compare coronal, middle, and apical sections in the groups, repeated measures ANOVA was employed with the use of Greenhouse-Geisser correction if necessary. Statistically significant differences between sections were analyzed using the Bonferroni test. The significance level was considered to be *p* < 0.01.

## 4. Results

### 4.1. SEM Examination

**Figure 1:**
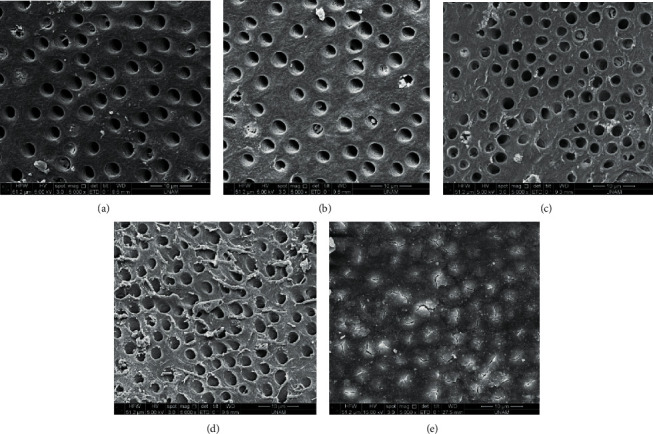
Representative scanning electron microscopy images of the removal of smear layer out of root canal walls. (a) 17% EDTA and distilled water (group 1). (b) 17% EDTA, distilled water, and 2% CHX (group 2). (c) 7% MA and distilled water (group 3). (d) 7% MA, distilled water, and 2% CHX (group 4). (e) 5.25% NaOCl and distilled water (group 5). Magnification: 5000x.

After the irrigation protocol, two roots out of each of the groups were evaluated by SEM to confirm smear layer removal. In group 5 (5.25% NaOCl), no open dentinal tubules were present. In other groups, smear layer removal was observed to various degrees as shown in Figure 1.

### 4.2. CLSM Evaluation

Tables 1–3 show both the percentage of penetration and maximum penetration (*μ*m) depth of coronal, middle, and apical thirds.

A statistically significant difference between sealers was observed in the coronal, middle, and apical thirds. Maximum penetration was observed with AH Plus, EndoRez, and Tech BioSealer Endo sealers in each sections (coronal, middle, and apical thirds), respectively.

**Figure 2:**
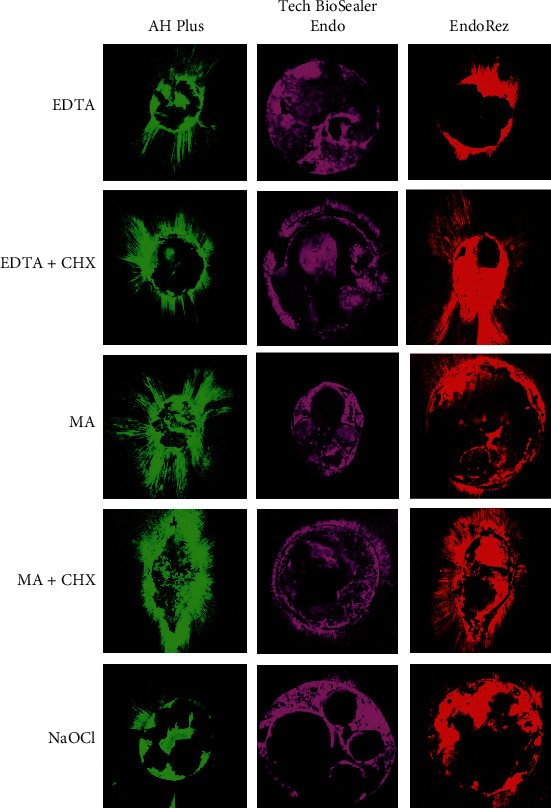
Representative CLSM images of the penetration of sealant around the walls of the root canal in coronal thirds (magnification, 10x).

**Figure 3:**
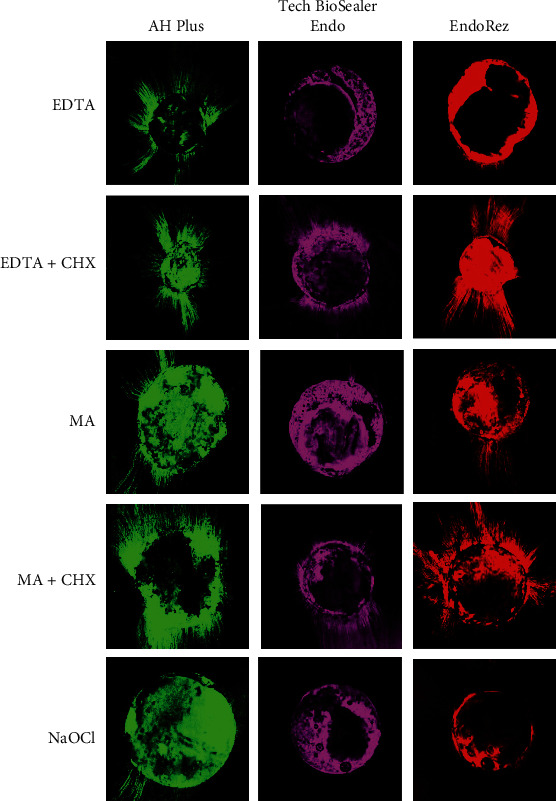
Representative CLSM images of the penetration of sealant around the walls of the root canal in middle thirds (magnification, 10x).

**Figure 4:**
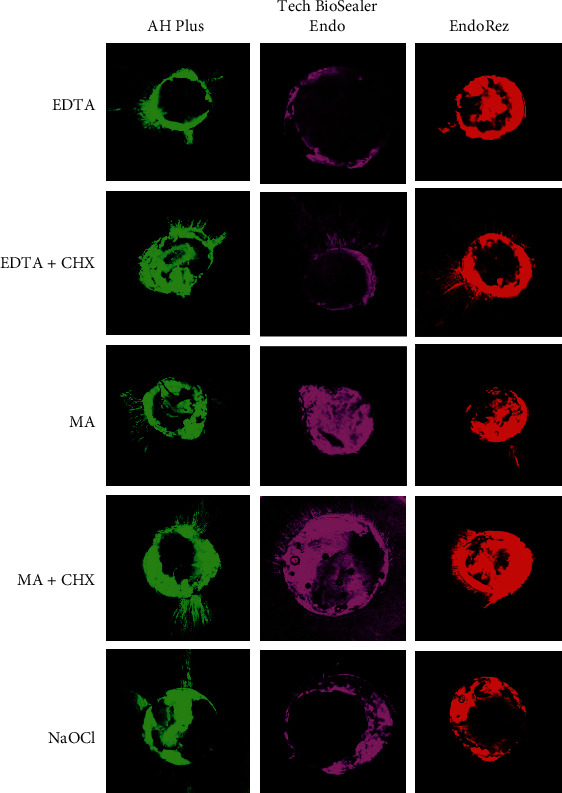
Representative CLSM images of the penetration of sealant around the walls of the root canal in apical thirds (magnification, 10x).

In Figures 2–4, simulated patterns of penetration of the sealant around the root canal walls in the coronal, middle, and apical thirds are shown.

Group 5 had a significantly lower penetration percentage and max. sealant depth value than the other groups. Considering the coronal sections, group 2 and group 4 showed a higher penetration percentage and max. sealant depth value than group 1 and group 3, except for group 4A which showed lower maximum penetration depth values than group 3A.

## 5. Discussion

Root canal therapy is aimed at clearing the root canal system off the microorganisms and at restraining recontamination [19]. Root canal sealers increase the contact area between gutta-percha and the root dentin, and thus, the root canal system is better occluded [20, 21]. Hence, it was aimed by this *in vitro* study to make an assessment on the penetration of three types of root canal sealants into dentinal tubules following differing irrigation processes performed using confocal laser scanning microscopy. Many different investigations have defined that CLSM represents a versatile method in the study of dentin and/or cement interface and the vitality of bacteria. CLSM provides adequate information on the adaptation and/or distribution of the sealer within the root canal system and dentinal tubules, and it allows the visualization of materials with various compositions. Moreover, the CLMS has been defined as a nondestructive technique since it allows the use of the same sample for additional analysis [22, 23]. As our study included a large number of samples and to avoid confusion in examining tubule penetration, we decided to save CLSM images in “different colors.” On the other hand, CLSM images in “different colors” may help the reader for better understanding. The study found no significant difference between chelating agents, but 2% CHX solution used after the chelating agents significantly improved sealer penetration in each segments (coronal, middle, and apical thirds). Garcia-Godoy et al. [24] showed that EDTA and MTAD solutions work well in the removal of the smear layer and that, however, each solution all by itself causes collapse of the dentinal matrix preventing the infiltration of sealant. This phenomenon may be the reason for samples treated with chelating solutions (groups 1 and 3) showing lower tubule penetration than samples additionally treated with CHX (groups 2 and 4). Oliveira et al. [25] reported that 1% NaOCl and 2% CHX solutions caused a significant reduction in dentinal microhardness. CHX irrigation in group 2 and group 4 may have caused a greater reduction in dentin microhardness, thus allowing the sealers to penetrate the dentinal tubules more easily through the obturation forces created by lateral compaction. Therefore, the physicochemical effect of CHX on dentinal matrix and the extra irrigation volume of the CHX groups may explain this component of our study.

Tuncer et al. [26] conducted an analysis of the effect of maleic acid on sealer penetration using AH 26 root canal sealer and found no significant difference between 7% MA, 17% EDTA, and 10% citric acid. In our study, 7% MA and 17% EDTA were used as chelating agents, where no significant difference was revealed between the respective groups (group 1 vs. group 3, group 2 vs. group 4).

Also in our study, epoxy AH Plus with resin base produced more favorable results than methacrylate resin-based EndoRez. Results in the literature differ when comparing penetration of these two root canal sealants into dentinal tubule. Chandra et al. [27] compared the penetration level of four resin-based sealers into dentinal tubule. RealSeal had the best tubule penetration values, and AH Plus showed better tubule penetration results than EndoRez sealer. They noted that the penetration of sealer into dentinal tubules is caused by capillary movement of the sealers and that the setting time of the sealers plays a critical role in tubule penetration. Based on this idea, they concluded that the tubule penetration results for AH Plus may be superior because of its longer setting time than light-cured EndoRez. Chadha et al. [28] found that the resin-based sealers EndoRez and Epiphany had better tubule penetration values than epoxy-resin based AH Plus. Their study left the root canals moist and used EndoRez sealer with resin-coated gutta-percha. The hydrophilic behaviour of EndoRez may therefore explain these results. The results in our study could be attributed to the superior physical properties of the AH Plus sealer. AH Plus paste shows high fluidity [29], low solubility, long curing time, less shrinkage during polymerisation, and expansion within the humid root canal environment [30].

Tech BioSealer Endo root canal sealer showed the lowest tubule penetration values in our study. Tech BioSealer Endo contains white Portland cement, bismuth dioxide as a radiopacity enhancer, anhydrite, and sodium fluoride [16]. Coomaraswamy et al. [31] reported that the bismuth oxide causes porosity, lessens durability, and shortens the lifespan of the material. These physical properties of the material may explain its poor tubule penetration values. Bortolini et al. [32] analyzed the penetration of Endo CPM, AH Plus, and EndoRez sealers into the tubule and reported that Endo CPM sealer showed the least dentinal tubule penetration. The particle size of the sealer materials is an important factor in dentinal tubule penetration. Dentinal tubules are thought to be between 2 and 5 *μ*m in diameter. The particle diameter of bismuth oxide is 10-30 *μ*m or greater [33], so preventing the sealer penetrating into dentinal tubules. Viapiana et al. [34] showed that experimental sealers that contain the smaller micro/nanoparticulates zirconium and niobium instead of bismuth dioxide may penetrate into dentinal tubules. On the other hand, the apical region of the root is where one can find more anatomical complexities such as accessory canals, apical delta and isthmus. Moreover, areas of atubular (sclerotic) dentine are more common in the apical third [17, 35, 36]. In our study, the depth and area of sealer penetration were reduced in the apical third, which can be explained by the smaller number of tubules and their smaller diameter and even tubular obliteration in this region.

## 6. Conclusion

Within the limits of the present study, Portland cement-based Tech BioSealer Endo showed the least dentinal tubule penetration while epoxy resin-based AH Plus showed better values then the resin-based EndoRez sealer. Tech BioSealer Endo root canal sealer showed unfavorable results using the lateral condensation method. The final irrigation regimens had statistically significant effect of sealer penetration values. When 7% MA and 17% EDTA solutions were used as final irrigants, no significant difference was observed regarding sealer penetration into dentinal tubules. The last irrigation with 2% CHX, after using chelating agents, improved sealer penetration percentages and had a positive effect on sealer penetration depth. Further studies are needed to evaluate sealer penetration with other obturation methods and final irrigation protocols.

## Figures and Tables

**Table 1:** Percentage of penetration and maximum penetration (*μ*m) depth of coronal sections (mean ± SD).

			Percentage of penetration (%)		Maximum penetration depth (*μ*m)	
			Means	Standard deviation	Means	Standard deviation
AH plus	EDTA	G1A	81.67^a^	15.76	722.22^ab^	232.77
	EDTA+CHX	G2A	87.24^a^	9.80	770.75^ab^	171.34
	MA	G3A	77.46^a^	12.91	844.67^b^	133.20
	MA+CHX	G4A	88.33^a^	7.70	605.96^ac^	129.02
	NaOCl	G5A	24.76^b^	8.48	490.18^c^	147.64

EndoRez	EDTA	G1E	58.75^a^	19.03	289.37^a^	149.15
	EDTA+CHX	G2E	76.29^a^	6.19	764.93^b^	93.33
	MA	G3E	56.73^a^	14.59	185.47^a^	80.02
	MA+CHX	G4E	73.16^a^	16.86	512.12^c^	171.15
	NaOCl	G5E	15.60^b^	9.35	69.83^d^	33.03

Tech BioSealer Endo	EDTA	G1T	5.02^a^	2.30	35.57^a^	14.50
	EDTA+CHX	G2T	53.38^b^	16.45	130.74^b^	105.93
	MA	G3T	9.33^a^	4.48	51.90^a^	41.40
	MA+CHX	G4T	66.08^b^	14.93	136.84^b^	91.05
	NaOCl	G5T	6.79^a^	3.62	42.78^a^	22.59

For each sealer, letters next to the values indicate whether there is a statistical difference according to the results of two-way analysis of variance. The same letters mean no statistical difference among groups. SD: standard deviation.

**Table 2:** Percentage of penetration and maximum penetration (*μ*m) depth of middle sections (mean ± SD).

			Percentage of penetration (%)		Maximum penetration depth (*μ*m)	
			Means	Standard deviation	Means	Standard deviation
AH plus	EDTA	G1A	56.59^a^	11.56	366.79^ab^	115.87
	EDTA+CHX	G2A	73.78^b^	8.83	535.56^b^	123.28
	MA	G3A	53.23^a^	7.02	526.13^b^	159.41
	MA+CHX	G4A	67.85^b^	13.25	344.51^a^	139.19
	NaOCl	G5A	20.54^c^	7.06	265.76^a^	118.85

EndoRez	EDTA	G1E	39.26^a^	8.70	104.64^a^	97.85
	EDTA+CHX	G2E	59.19^b^	16.50	596.23^b^	246.83
	MA	G3E	42.12^a^	15.83	249.59^c^	166.60
	MA+CHX	G4E	60.26^b^	10.01	259.26^c^	89.13
	NaOCl	G5E	11.23^c^	7.25	86.38^a^	71.05

Tech BioSealer Endo	EDTA	G1T	3.37^a^	1.84	17.54^a^	3.96
	EDTA+CHX	G2T	33.82^b^	16.32	88.49^b^	46.25
	MA	G3T	6.97^a^	2.88	24.52^a^	9.61
	MA+CHX	G4T	52.91^c^	17.90	120.91^c^	74.70
	NaOCl	G5T	5.34^a^	1.18	27.15^a^	13.38

**Table 3:** Percentage of penetration and maximum penetration (*μ*m) depth of apical sections (mean ± SD).

			Percentage of penetration (%)		Maximum penetration depth (*μ*m)	
			Means	Standard deviation	Means	Standard deviation
AH plus	EDTA	G1A	35.53^a^	11.92	123.31^a^	49.57
	EDTA+CHX	G2A	54.67^b^	4.49	211.03^a^	90.08
	MA	G3A	35.36^a^	6.91	190.79^a^	81.15
	MA+CHX	G4A	45.57^b^	4.36	129.83^a^	77.42
	NaOCl	G5A	15.33^c^	6.37	130.91^a^	67.48

EndoRez	EDTA	G1E	20.46^a^	10.11	37.06^a^	16.08
	EDTA+CHX	G2E	45.31^c^	12.72	180.48^b^	154.42
	MA	G3E	24.88^ab^	4.66	72.01^c^	94.88
	MA+CHX	G4E	37.82^b^	11.75	120.28^c^	39.44
	NaOCl	G5E	11.79^a^	8.41	24.00^a^	13.65

Tech BioSealer Endo	EDTA	G1T	3.34^a^	2.40	14.16^a^	4.54
	EDTA+CHX	G2T	20.27^b^	11.58	40.07^b^	29.53
	MA	G3T	4.36^a^	1.62	16.12^a^	9.67
	MA+CHX	G4T	27.99^b^	8.30	40.94^b^	18.21
	NaOCl	G5T	3.61^a^	1.44	17.25^a^	3.23

## Data Availability

All data of the study are presented in the article and are available on request by contacting the corresponding author.
